# Use of high throughput T cell receptor (TCR) DNA sequencing to characterize T cell infusion products and track clonal expansion *in vivo*

**DOI:** 10.1186/2051-1426-2-S3-P143

**Published:** 2014-11-06

**Authors:** Madeleine Strohl, Hallie Graor, Mei Zhang, Isabelle Rivers-McCue, John Ammori, Julian Kim

**Affiliations:** 1 Case Western Reserve University School of Medicine, Cleveland, OH, USA; 2 Division of Surgical Oncology, University Hospitals Case Medical Center, Cleveland, OH, USA

## Background

During the process of T cell priming to peptide antigens, the T cell receptor (TCR) undergoes gene rearrangements that result in a peptide-binding region (complementarity determining region, CDR) which can act as a unique molecular fingerprint for similar T cell clonotypes. The purpose of this study was to assess high-throughput TCR DNA sequencing to analyze qualitative and quantitative measures of human T cell clonotype expansions both in vitro and in vivo.

## Methods

Human lymph nodes derived from patients with melanoma (melanoma-draining lymph node cells, MDLN) were culture-activated using either anti-CD3/CD28 beads and IL-2 100 IU/ml alone or in combination with anti-VEGF neutralizing antibody, for 14 days. The two resultant MDLN cultures were compared for overall T cell expansion, cell surface phenotype, intracellular cytokine staining and high-throughput TCR DNA sequencing (ImmunoSEQ, Adaptive Biotechnologies). In addition, both TDLN cell cultures were injected intravenously into SCID mice bearing A375 melanoma xenografts and bone marrow was harvested to assess for persistence of transferred human melanoma MDLN cells.

## Results

Overall T cell expansion and cell surface phenotype of MDLN cell cultures was similar. MDLN cells cultured in the presence of anti-VEGF neutralizing antibody demonstrated higher baseline levels of intracellular interferon-γ. TCR sequencing analysis comparing day 0 versus day 14 cultured MDLN cells demonstrated approximately 10% shared T cell clonotypes following culture activation with anti-CD3/CD28 beads and IL-2. As a point of reference, the proportion of shared T cell clonotypes between two different patient MDLN cells was approximately 0.6%. Addition of anti-VEGF antibody during culture of MDLN cells resulted in common T cell clonotypes of approximately 1% (day 0 versus day 14), confirming the expansion of molecularly distinct T cell clonotypes. Human MDLN cells infused into SCID mice bearing A375 melanoma xenografts demonstrated persistence of T cell clonotypes in the bone marrow several weeks after infusion. It is notable that none of the low frequency T cell clonotype expansion differences between samples could be readily identified using VDJ gene usage or CDR3 length analysis.

## Conclusions

This study demonstrates that CDR3 sequence data that is quantitative and recognizes low frequency T cell clonotypes is more sensitive than V gene usage and CDR3 length analysis. In addition, CDR3 sequencing may provide utility in the qualitative assessment of T cells both in vitro and in vivo.

